# Periodontal Phenotype Thickening Using Sticky Bone and Platelet-Rich Fibrin: A Case Report

**DOI:** 10.7759/cureus.91968

**Published:** 2025-09-10

**Authors:** Maria Burgos-Anaya, Jaime E Plazas Román, Alejandra Herrera, Jonathan Harris-Ricardo, Antonio Diaz, Carlos M Ardila

**Affiliations:** 1 Dentistry, Corporación Universitaria Rafael Núñez, Cartagena, COL; 2 Dentistry, Universidad de Cartagena, Cartagena, COL; 3 Dentistry, Universidad del Sinú, Section Cartagena, Cartagena, COL; 4 Dentistry, Universidad Metropolitana, Barranquilla, COL; 5 Basic Sciences, Biomedical Stomatology Research Group, Faculty of Dentistry, University of Antioquia, Medellin, COL

**Keywords:** connective tissue graft, guided bone regeneration, mucogingival surgery, periodontal treatment, platelet-rich fibrin

## Abstract

Periodontal phenotype thickening is essential in periodontal and peri-implant plastic surgery, since thin phenotypes are strongly associated with increased risk of gingival recession, marginal bone loss, and reduced long-term stability. While connective tissue grafts have traditionally been regarded as the gold standard, they present significant limitations related to donor site morbidity and surgical complexity. Biologically active autologous biomaterials such as platelet-rich fibrin (PRF) and sticky bone have recently emerged as promising minimally invasive alternatives. We report a case of a 50-year-old systemically healthy female patient with a thin periodontal phenotype in the anterior mandibular region. A regenerative approach was performed using particulate xenograft mixed with liquid PRF to create sticky bone, complemented by an advanced PRF membrane placed as a biological matrix, followed by primary closure. Clinical evaluation at nine months demonstrated substantial periodontal phenotype thickening, increased keratinized tissue volume, preservation of alveolar ridge contour, and stable integration of the graft without complications. The patient reported minimal postoperative discomfort and exhibited excellent functional and esthetic outcomes. This case highlights the effectiveness of PRF combined with sticky bone as a biologically driven, predictable, and less invasive strategy for periodontal phenotype modification, offering enhanced tissue integration, reduced morbidity, and long-term stability compared to conventional grafting techniques.

## Introduction

Periodontal phenotype, previously termed gingival biotype, refers to the morphology of periodontal tissues, particularly the thickness of keratinized gingiva, the amount of supracrestal soft tissue, and the underlying bone architecture [1]. Its classification into thin or thick phenotype has significant clinical implications, especially in periodontal surgery, implantology, and oral rehabilitation [2]. The thin phenotype is associated with a greater tendency toward gingival recessions, root exposure, marginal bone loss, and less predictable tissue behavior during surgical and restorative procedures [3].

In response to these challenges, periodontal phenotype thickening has emerged as a key therapeutic strategy to improve surgical prognosis, reduce recession risk, and achieve more stable long-term aesthetic results [4]. Among the techniques used for this purpose, the combined use of platelet-rich fibrin (PRF) and sticky bone stands out, a technique that simultaneously allows soft tissue volume augmentation and guided bone regeneration [5,6].

Platelet-rich fibrin (PRF) is an autologous biomaterial obtained through centrifugation without anticoagulants, generating a three-dimensional matrix rich in platelets, leukocytes, and growth factors such as PDGF, VEGF, and TGF-β1 [7]. These factors promote angiogenesis, cell proliferation, and osteoblastic and fibroblastic differentiation, favoring accelerated healing of soft and hard tissues [8]. Recent studies have demonstrated that injectable PRF (i-PRF) is particularly effective for periodontal phenotype thickening, showing comparable results to connective tissue grafts with lower morbidity [9,10].

Sticky bone is a cohesive mass obtained by mixing particulate bone graft (xenograft or allograft) with liquid-phase PRF [11]. This compound acts as a moldable, biologically active, and highly stable graft that improves intraoperative manipulation, spatial retention of the graft, and controlled bone regeneration [12]. Its integration in procedures where thin phenotype can compromise soft tissue stability has been key to achieving effective and prolonged three-dimensional support [13]. The combination of PRF and sticky bone for periodontal phenotype thickening has proven particularly useful in guided bone regeneration (GBR) surgeries, peri-implant procedures, and multiple recession treatments, where soft tissue support and bone preservation are fundamental [14,15].

This study aimed to describe the clinical application and evaluate the outcomes of periodontal phenotype thickening using a combination of PRF and sticky bone in a patient with a thin periodontal phenotype, demonstrating the technique's effectiveness as a minimally invasive alternative to traditional approaches.

## Case presentation

A 50-year-old systemically healthy female patient presented for periodontal treatment for reconstructive and esthetic purposes. The clinical objective was to achieve periodontal phenotype thickening using sticky bone and platelet-rich fibrin (PRF), thereby improving soft tissue support and long-term stability. Written informed consent was obtained from the patient for the publication of clinical data and images.

**Figure 1:**
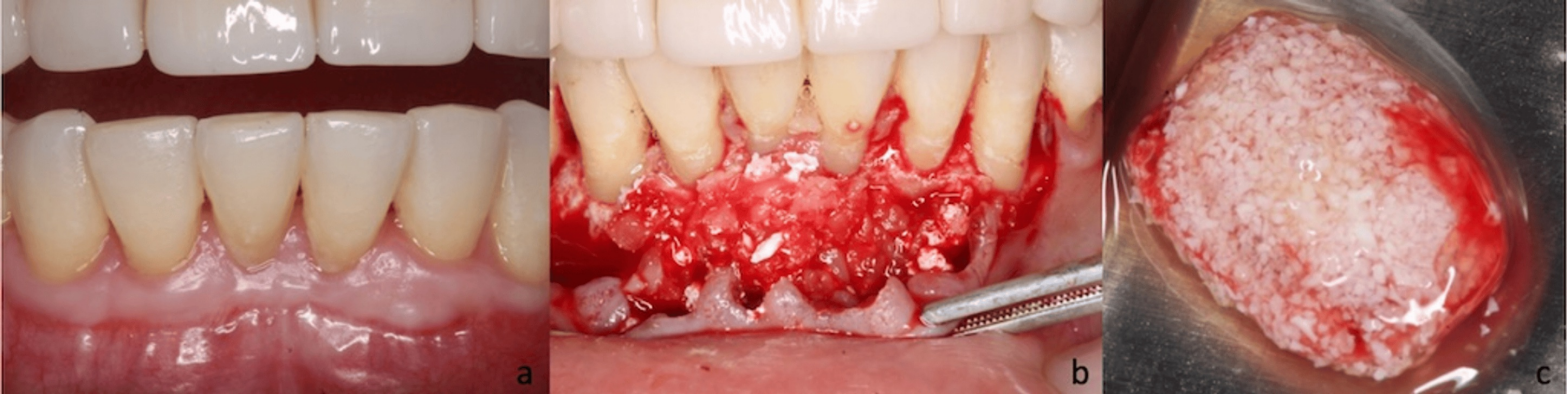
Clinical evaluation and graft preparation. (a) Initial clinical view of the anterior mandibular region, showing a thin periodontal biotype with limited buccal volume and restricted keratinized gingiva. (b) Intraoperative image after full-thickness flap elevation, exposing the horizontal bone defect. A thin buccal plate and a need for tissue thickening are observed. (c) Preparation of bovine particulate xenograft hydrated with autologous blood. This will subsequently be mixed with liquid platelet-rich fibrin to form "sticky bone".

Initial clinical evaluation revealed a thin periodontal phenotype, characterized by limited keratinized gingiva in the anterior mandibular region (Figure 1a). Baseline measurements showed a gingival thickness of 0.8 mm measured with a periodontal probe. After disinfection, bilateral mental nerve block and infiltrative buccal and lingual anesthesia were performed. A full-thickness flap was raised with careful tissue elevation to expose the bone bed, revealing a horizontal defect with insufficient buccal thickness (Figure 1b). The regenerative biomaterial was prepared using particulate xenograft bone hydrated with the patient's autologous blood, which was mixed with liquid PRF to form sticky bone (Figure 1c). This graft, rich in growth factors and with cohesive consistency, adapted perfectly to the treated defect.

**Figure 2:**
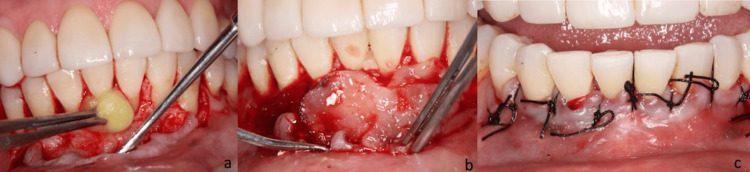
Graft application and surgical closure. (a) Placement of platelet-rich fibrin membrane (A-PRF) over the recipient area, acting as a bioactive matrix to promote angiogenesis, control inflammation, and enhance regeneration. (b) Insertion of "sticky bone" graft, composed of particulate xenograft mixed with liquid PRF. Its excellent adaptability and three-dimensional stability over the defect are observed. (c) Primary tissue closure using simple interrupted sutures with 4-0 surgical silk, ensuring tension-free flap coaptation for primary intention healing.

A platelet-rich fibrin membrane (A-PRF type) was placed, acting as biological coverage and healing matrix over the bone graft, promoting angiogenesis and clot stabilization (Figure 2a). The sticky bone was completely adapted to the defect, with excellent clinical manipulation and three-dimensional conformation of the desired volume (Figure 2b). Finally, primary closure was achieved using simple interrupted sutures with 4-0 silk thread, obtaining proper tissue coaptation without tension (Figure 2c).

Postoperative medications included amoxicillin 500 mg every 8 hours for seven days, ibuprofen 400 mg every 8 hours for five days, and chlorhexidine 0.12% rinses twice daily for 15 days. Postoperative instructions included soft diet for 48 hours and avoiding brushing in the operated area during the first week.

Follow-up appointments were scheduled at 15 days, one month, three months, six months, and nine months postoperatively. Clinical evaluation included assessment of inflammation signs, tissue stability, gingival thickness measurement with periodontal probe, and photographic documentation.

**Figure 3:**
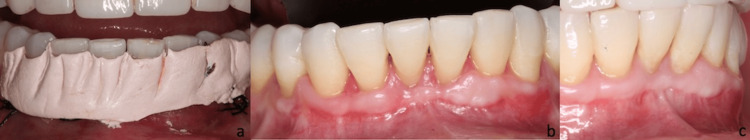
Postoperative follow-up and clinical outcomes. (a) Recording through impression for the study model a few days after the procedure, allowing evaluation of adaptation and achieved volume. (b) Clinical result at one month, evidencing periodontal phenotype thickening with increased volume in buccal soft tissues and a healthy appearance. (c) Lateral view of the treated area confirming increased keratinized tissue thickness and stable integration without inflammatory signs.

During postoperative follow-up, healing was evaluated through study models (Figure 3a). Results at nine months showed notable periodontal phenotype thickening with gingival thickness increasing from 0.8 mm to 1.6 mm, representing a 100% increase in tissue volume, increased soft tissue volume, and marginal stabilization without signs of inflammation or dehiscence (Figures 3b, 3c).

## Discussion

Periodontal phenotype thickening has become established as an essential intervention in periodontal and peri-implant plastic surgery, as it improves soft tissue resistance to trauma, enhances tissue healing, and contributes to long-term peri-implant maintenance [2]. Traditionally, the subepithelial connective tissue graft has been considered the gold standard for thin biotype modification due to its high clinical predictability and volumetric stability [3]. However, its use involves limitations such as the need for a second surgical site and increased postoperative morbidity.

The present case aligns with findings reported in recent literature on the use of PRF for periodontal phenotype thickening. Manasa et al. evaluated the efficacy of i-PRF for modifying periodontal phenotypes in a randomized controlled split-mouth clinical trial, demonstrating significant increases in gingival thickness (from 0.85±0.23 mm to 1.42±0.35 mm) after three months [9]. The present case showed superior results with a 100% increase in gingival thickness at nine months. Similarly, Ozsagir et al. reported favorable results combining i-PRF with microneedling for periodontal thickening, observing statistically significant improvements in tissue thickness at six-month follow-up [10].

Recent studies have compared different approaches for thin periodontal phenotype management, finding that minimally invasive methods using autologous biomaterials are effective for increasing gingival thickness and keratinized tissue width [14]. This suggests that PRF represents a biologically favorable and cost-effective alternative.

Unlike connective tissue grafts, which require an additional donor site and present greater postoperative morbidity, the use of PRF and sticky bone offers significant advantages. It was documented that PRF allows effective periodontal biotype thickening with fewer complications and comparable clinical results [4]. Meta-analyses reinforce these findings, evidencing that PRF application significantly enhances dental implant stability and soft tissue volume improvement, directly influencing esthetic and functional outcomes [6].

The sticky bone used in this case represents an evolution in bone regeneration techniques. Recent clinical trials have demonstrated that sticky bone with i-PRF application shows improved results in managing periodontal intrabony defects compared to conventional techniques [11]. This approach is highly effective in guided bone regeneration procedures, especially when combined with resorbable collagen membranes [6].

The combination of PRF with particulate bone grafts not only improves soft and hard tissue volume but also favors better tissue integration and accelerates healing [11]. Miron et al. described in their systematic review that PRF use in periodontal intrabony defects was significantly effective, with additional benefits when combined with bone grafts [8].

A recent systematic review and meta-analysis that included multiple randomized clinical trials demonstrated that the use of PRF, combined with open flap debridement, PRF + metformin, and PRF + bone graft, significantly reduced probing depth and improved clinical attachment levels and radiographic bone fill [8]. Furthermore, a recent clinical trial has demonstrated that leukocyte-platelet-rich fibrin membranes show significant efficacy in immediate postextraction implant placement, supporting the biological rationale for PRF use in tissue regeneration procedures [12].

Study limitations

We acknowledge several limitations of this study. The absence of radiographic documentation limits the assessment of bone graft integration. Future cases should include periapical radiographs or cone-beam computed tomography for comprehensive evaluation. Additionally, the single-case design limits the generalizability of results, and controlled clinical trials are needed to establish standardized protocols.

Although results are promising, it is important to consider the limitations of the presented case. Recent systematic reviews emphasize that while PRF significantly improves periodontal tissue regeneration through the release of key growth factors such as PDGF and TGF-β, more controlled clinical trials with long-term follow-ups are required to standardize protocols and fully validate its efficacy against conventional techniques [8,13].

The present case incorporates innovative elements documented in recent literature. Studies have described biological properties of novel bone blocks composed of PRF and deproteinized bovine bone mineral, demonstrating favorable characteristics for bone regeneration [11]. Recent reviews on optimized bone grafts highlight that the combination of xenografts with platelet concentrates represents the future of periodontal regeneration [15].

## Conclusions

This case demonstrates that the combination of PRF and sticky bone effectively achieves periodontal phenotype thickening with minimal invasiveness and reduced morbidity compared to traditional techniques. The 100% increase in gingival thickness observed at nine months, along with stable tissue integration without complications, supports this approach as a viable alternative to connective tissue grafts.

Current evidence supports integrating these biological therapies into a comprehensive, personalized, and minimally invasive treatment approach that aims to maximize both clinical and esthetic success. Nevertheless, despite their promising outcomes, there remains a pressing need for well-designed, controlled clinical trials with long-term follow-ups to establish standardized protocols and confirm their long-term efficacy when compared to traditional methods.
